# É Hora de Incluir o Treinamento de Equilíbrio nos Programas de Reabilitação Cardíaca de Pacientes com Insuficiência Cardíaca com Fração de Ejeção Preservada

**DOI:** 10.36660/abc.20200157

**Published:** 2020-05-12

**Authors:** Fernando Ribeiro

**Affiliations:** 1 Instituto de Biomedicina Escola Superior de Saúde Universidade de Aveiro Aveiro Portugal Instituto de Biomedicina (iBiMED) - Escola Superior de Saúde - Universidade de Aveiro,Aveiro – Portugal

**Keywords:** Insuficiência Cardíaca/fisiopatologia, Reabilitação Cardíaca, Volume Sistólico, Débito Cardíaco, Hospitalização

O ônus da insuficiência cardíaca exerce um impacto pessoal, social e econômico significativo, não apenas nos pacientes e suas famílias, mas também na sociedade (incluindo os sistemas de saúde). A insuficiência cardíaca é uma condição crônica e progressiva que afeta um grande número de indivíduos em todo o mundo (> 37,7 milhões de casos estimados em 2010).^1^ É caracterizada por sintomas típicos (por exemplo, falta de ar e fadiga) que podem ser acompanhados por sinais (por exemplo, edema periférico) causados por anormalidades cardíacas – estruturais e /ou funcionais –, resultando em débito cardíaco reduzido e/ou pressões intracardíacas elevadas em repouso ou durante o estresse.^2^ A insuficiência cardíaca foi categorizada em insuficiência cardíaca com fração de ejeção preservada (ICFEp), intermediária (ICFEm) e reduzida (ICFEr), de acordo com a fração de ejeção do ventrículo esquerdo.^2^

A insuficiência cardíaca com fração de ejeção preservada tornou-se um fenótipo cada vez mais reconhecido; apesar de ser considerada uma condição que afeta principalmente indivíduos idosos, representa aproximadamente metade de todos os casos de insuficiência cardíaca.^3 , 4^ Apesar das terapias farmacológicas e dos dispositivos disponíveis, o prognóstico dos pacientes com insuficiência cardíaca, qualidade de vida e sobrevida em 5 anos permanecem ruins^5^ e similares em todas as categorias de insuficiência cardíaca.^4 - 6^

A insuficiência cardíaca é uma causa frequente de hospitalização, principalmente em idosos.^7^ Indivíduos idosos com insuficiência cardíaca hospitalizados por causas cardiovasculares, como insuficiência cardíaca descompensada aguda, são geralmente frágeis e apresentam baixa qualidade de vida e comprometimentos graves em vários componentes da aptidão física, incluindo capacidade de exercício, força muscular, equilíbrio e mobilidade.^8^ Essas deficiências podem ajudar a explicar por que as hospitalizações de pacientes com ICFEp estão frequentemente relacionadas a causas não cardiovasculares.^9^ Menor aptidão física, comprometimento do equilíbrio e da mobilidade funcional, e o uso de alguns medicamentos (por exemplo, digoxina) aumenta o risco de quedas em pacientes idosos com insuficiência cardíaca. Foi relatada não apenas uma associação entre insuficiência cardíaca e aumento do risco de queda, mas também uma taxa de queda muito maior (43%) em pacientes com insuficiência cardíaca em comparação com pacientes com doença arterial coronariana (34%) ou diabetes mellitus (28%).^10 , 11^

A aptidão física é um construto de atributos relacionados à saúde (composição corporal, resistência cardiorrespiratória, flexibilidade, resistência muscular, força) e relacionados ao desempenho desportivo (equilíbrio, agilidade e coordenação, velocidade e tempo de reação) que se refere à habilidade de nossos sistemas corporais em trabalhar juntos de forma eficiente. Nesta edição da revista, Schmidt et al.^12^ exploram essa questão em uma coorte de pacientes idosos (média de idade: 76 ± 6 anos) com ICFEp. Os autores avaliaram a associação entre diferentes componentes da aptidão física – capacidade de exercício, força de preensão manual, equilíbrio dinâmico e mobilidade e composição corporal – e as dimensões da qualidade de vida de pacientes com ICFEp. Eles também avaliaram quais componentes da aptidão física eram independentemente associados à qualidade de vida relacionada à saúde. Os autores realizaram um estudo transversal com uma amostra de conveniência de 24 pacientes (17 mulheres e 7 homens), 79% deles com classe funcional II da *New York Heart Association* (NYHA) (n = 19) e apenas quatro pacientes (21%) com classe funcional III da NYHA. Eles encontraram uma associação significativa entre a distância atingida no teste de caminhada de 6 minutos (TC6M) (capacidade de exercício) e o escore no teste *8-foot Up and Go* (equilíbrio dinâmico e mobilidade) com o escore total e a dimensão física do *Minnesota Living With Heart Failure Questionnaire* (qualidade de vida relacionada à saúde), mas apenas o equilíbrio dinâmico e a mobilidade foram concomitantemente associados à dimensão emocional. Curiosamente, apenas o desempenho no teste *8-foot Up and Go* (equilíbrio dinâmico e mobilidade) foi associado à qualidade de vida – escore total, dimensões físicas e emocionais – após o ajuste para idade, sexo e classe funcional da NYHA. Os pacientes com melhor equilíbrio também relataram melhor qualidade de vida. Neste estudo, o pico de consumo de oxigênio durante o teste de exercício cardiopulmonar não foi avaliado e, portanto, não é possível determinar se existe uma associação entre uma medida máxima ou limitada por sintomas da capacidade de exercício e qualidade de vida, bem como se o equilíbrio ainda está associado à qualidade de vida ao controlar também o consumo máximo de oxigênio. Apesar do pequeno tamanho amostral predominantemente composto por mulheres (71%) com comprometimento funcional leve e a amostragem por conveniência, este estudo gerou dados interessantes que podem ser utilizados para informar futuros e maiores estudos nessa área. Uma análise secundária dos estudos RELAX e NEAT-HFpEF, recentemente publicada,^13^ avaliou diferenças de sexo na capacidade de exercício (TC6M) e na qualidade de vida ( *Minnesota Living With Heart Failure Questionnaire* ) em 323 pacientes com ICFEp (158 homens e 165 mulheres) e encontrou diferentes determinantes da qualidade de vida entre mulheres e homens. Curiosamente, a qualidade de vida foi associada à disfunção diastólica, cardiopatia isquêmica e capacidade de exercício em homens, enquanto em mulheres, somente o índice de massa corporal e a idade predizem a qualidade de vida.

O equilíbrio dinâmico e a mobilidade poderiam ser um dos determinantes da qualidade de vida em mulheres com ICFEp? O estudo de Schmidt et al.,^12^ deu alguns esclarecimentos a respeito dessa questão, pois recrutou uma amostra composta predominantemente por mulheres (71%), utilizaram as mesmas ferramentas para avaliar a qualidade de vida e a capacidade de exercício e concluíram que o equilíbrio dinâmico e a mobilidade superam a capacidade de exercício na avaliação da qualidade de vida em pacientes com ICFEp. Coletivamente, esses achados reforçam a importância da realização de estudos em mulheres com ICFEp para identificar determinantes de sua qualidade de vida.

As altas taxas de mortalidade, morbidade, rehospitalização cardiovascular e por insuficiência cardíaca e uso e custos de assistência médica associados ao aumento da prevalência de insuficiência cardíaca sinalizam claramente a necessidade de melhorar as estratégias de tratamento. O estudo de Schmidt et al.,^12^ certamente deixa o leitor com a sensação de que há um aspecto importante do cuidado médico em ICFEp que pode estar faltando em pacientes idosos. Como um preditor independente de qualidade de vida, todos os pacientes idosos devem ser testados quanto a equilíbrio dinâmico e mobilidade? Os déficits de equilíbrio são potencialmente tratáveis, e a identificação e o tratamento desses déficits podem melhorar a qualidade de vida dos pacientes. Uma investigação mais detalhada com maior tamanho de amostra é necessária para fortalecer ou refutar as conclusões de Schmidt et al.,^12^ e ajudar os médicos a decidir se testam ou não o equilíbrio diariamente.

O estudo de Schmidt et al.,^12^ ao sugerir o equilíbrio dinâmico e a mobilidade como os determinantes mais importantes da qualidade de vida (dimensões física e emocional), levanta também outra questão pertinente: é hora de incluir o treinamento de equilíbrio nos programas de reabilitação cardíaca de pacientes com ICFEp? A reabilitação cardíaca baseada no exercício é uma recomendação da classe 1A para pacientes com insuficiência cardíaca;^2^ em pacientes com ICFEp os benefícios são multidimensionais; por exemplo, um programa de reabilitação cardíaca baseado em exercício melhora a capacidade de exercício, a função diastólica e a qualidade de vida.^14 - 16^ Entretanto, os programas tradicionais de reabilitação cardíaca não abordam completamente as deficiências funcionais de múltiplos domínios, comuns em pacientes idosos com ICFEp, particularmente aquelas relacionadas a equilíbrio e mobilidade funcional. A resposta à pergunta acima mencionada pode ser dada em estudos que avaliem o impacto de programas de reabilitação cardíaca de múltiplos domínios, projetados para também melhorar o equilíbrio e a mobilidade funcional (além de outros objetivos, como melhorar a capacidade de exercício) administrados por uma equipe multidisciplinar; e avaliar se um programa que engloba exercícios específicos de equilíbrio e mobilidade funcional, além de exercícios aeróbicos e de resistência, é mais eficaz para melhorar o equilíbrio e a qualidade de vida, diminuir o risco e a taxa de quedas e reduzir as hospitalizações cardiovasculares e não cardiovasculares.

Em resumo, a atual contribuição de Schmidt et al.,^12^ nesta edição dos Arquivos Brasileiros de Cardiologia, aumenta a conscientização e fornece evidências para indicar a avaliação do equilíbrio dinâmico e da mobilidade em pacientes idosos com ICFEp. Entretanto, antes que isso seja implementado na rotina clínica, seus achados precisam ser fortalecidos em estudos futuros.
