# Supplementary material for: É Hora de Incluir o Treinamento de Equilíbrio nos Programas de Reabilitação Cardíaca de Pacientes com Insuficiência Cardíaca com Fração de Ejeção Preservada
Source: Arq Bras Cardiol. 2020 May 12;114(4):708–10. [Article in Portuguese] doi: 10.36660/abc.20200157 (PMC9744345; doi:10.36660/abc.20200157)
Supplement: Translation

# Translation: Time to Include Balance Training in the Cardiac Rehabilitation Programs of Patients with Heart Failure with Preserved Ejection Fraction

**Authors:** Fernando Ribeiro

**Affiliations:** 1 Portugal Instituto de Biomedicina (iBiMED) - Escola Superior de Saúde - Universidade de Aveiro, Aveiro – Portugal

**Keywords:** Heart Failure/physiopathology, Cardiac Rehabilitation, Stroke Volume, Cardiac Output, Hospitalization

The burden of heart failure exerts a significant personal, social and economic impact not only on patients and their families but also on society (including health care systems). Heart failure is a chronic, progressive condition affecting a huge amount of individuals worldwide (>37.7 million cases estimated in 2010).^1^ It is characterized by typical symptoms (e.g. breathlessness and fatigue) that may be accompanied by signs (e.g. peripheral edema) caused by cardiac abnormalities – structural and/or functional –, resulting in reduced cardiac output and/or elevated intracardiac pressures at rest or during stress.^2^ Heart failure has been categorized in heart failure with preserved (HFpEF), mid-range (HFmrEF) and reduced ejection fraction (HFrEF) according to the left ventricular ejection fraction.^2^

Heart failure with preserved ejection fraction has become an increasingly recognized phenotype; despite being primarily considered a condition that affects old-age individuals, it represents approximately half of all cases of heart failure.^3 , 4^ Despite the available pharmacological and device therapies, heart failure patients’ prognosis, quality of life, and 5-year survival remain poor^5^ and similar across all heart failure categories.^4 - 6^

Heart failure is a frequent cause of hospitalization, especially in older adults.^7^ Older adults with heart failure hospitalized for cardiovascular causes, namely acute decompensated heart failure, are usually frail and have a poor quality of life and severe impairments in several components of physical fitness including exercise capacity, muscle strength, balance and mobility.^8^ These impairments may help explain why the hospitalizations of patients with HFpEF are often related to non-cardiovascular causes.^9^ Lower physical fitness, namely balance and functional mobility impairments, and the use of some medications (e.g. digoxin) increases the risk of falling in old age patients with heart failure. It was reported not only an association between heart failure and increased fall risk but also a much higher fall rate (43%) in heart failure patients compared to patients with coronary artery disease (34%) or diabetes mellitus (28%).^10 , 11^

Physical fitness is a construct of health-related (body composition, cardiorespiratory endurance, flexibility, muscular endurance, power) and skill-related attributes (balance, agility, coordination, speed, reaction time) that refers to the ability of our body systems to work together efficiently. In this issue of the journal, Schmidt et al.^12^ explores this issue in an elderly (mean age 76 ± 6 years old) cohort of patients with HFpEF. The authors assessed the association between different components of physical fitness – exercise capacity, handgrip strength, dynamic balance and mobility, and body composition – and dimensions of quality of life of HFpEF patients. They also examined which physical fitness components were independently related to health-related quality of life. The authors carried out a cross-sectional study with a convenience sample of 24 patients (17 women and 7 men), 79% of them with a New York Heart Association (NYHA) functional class II (n=19) and only four patients (21%) with functional class III. They found a significant association between the 6-minute walk test (6MWT) distance (exercise capacity) and the score in the 8-foot up and go test (dynamic balance and mobility) with the total score and physical dimension score of the Minnesota Living With Heart Failure Questionnaire (health-related quality of life), but only dynamic balance and mobility was concomitantly associated with the emotional dimension. Interestingly, only the performance in the 8-foot up and go test (dynamic balance and mobility) was associated with quality of life – total score, physical and emotional dimensions – after adjusting for age, gender and NYHA functional class. Those patients with better balance also reported enhanced quality of life. In this study, peak oxygen consumption during cardiopulmonary exercise test was not assessed, and it is therefore not possible to determine whether there is an association between a maximal or symptom-limited measure of exercise capacity and quality of life, as well as whether balance is still associated with quality of life when also controlling for peak oxygen consumption. Despite the small sample size predominantly composed by women (71%) with mild functional impairment and the convenience sampling, this study generated interesting data that can be used to inform future and larger studies in this area. A secondary analysis of the RELAX and NEAT-HFpEF Trials recently published,^13^ assessed sex differences in exercise capacity (6MWT) and quality of life (Minnesota Living with Heart Failure Questionnaire) in 323 patients with HFpEF (158 men and 165 women) and found different determinants of quality of life between women and men. Interestingly, quality of life was associated with diastolic dysfunction, ischemic heart disease, and exercise capacity in men, while in women-only body mass index and age predicted quality of life. Could dynamic balance and mobility be one of the determinants of quality of life in women with HFpEF? The study of Schmidt et al.^12^ shed some light on this issue, as they recruited a sample composed predominantly by women (71%), used the same tools to assess the quality of life and exercise capacity, and concluded that dynamic balance and mobility outperforms exercise capacity in capturing HFpEF patients’ quality of life. Collectively, these findings reinforce the importance of carrying out studies in women with HFpEF to identify determinants of their quality of life.

The high mortality, morbidity, cardiovascular and heart failure readmission rates, and health care use and costs associated with the increase in heart failure prevalence clearly signal the need to improve treatment strategies. The study of Schmidt et al^12^ certainly leaves the reader with the feeling that there is an important aspect of HFpEF care that could be missing in old age patients. As an independent predictor of quality of life, should all old age patients be tested for dynamic balance and mobility? Balance deficits are potentially treatable, and identifying and treating such deficits may improve patients’ quality of life. Further investigation with larger sample size is needed to strengthen or refute Schmidt et al.^12^ conclusions and help clinicians decide whether to test or not balance daily.

The study of Schmidt et al.^12^ by suggesting dynamic balance and mobility as the most important determinant of quality of life (both physical and emotional dimensions), raises also another pertinent question: is it time to include balance training in the cardiac rehabilitation programs of patients with HFpEF? Exercise-based cardiac rehabilitation is a class 1A recommendation for heart failure patients;^2^ in patients with HFpEF the benefits are multi-dimensional, for instance, an exercise-based cardiac rehabilitation program improves exercise capacity, diastolic function, and quality of life.^14 - 16^ Nonetheless, traditional cardiac rehabilitation programs do not fully address the multi-domain functional impairments common in older patients with HFpEF, particularly balance and functional mobility impairments. The response to the above-mentioned question could be given in studies assessing the impact of multi-domain cardiac rehabilitation programs designed to also improve balance and functional mobility (in addition to other goals such as improve exercise capacity) administered by a multi-disciplinary team; and, assessing whether a program encompassing specific balance and functional mobility exercises in addition to aerobic and resistance exercise is more effective to improve balance and quality of life, decrease the risk and rate of fall, and to reduce cardiovascular and non-cardiovascular hospitalizations.

In summary, the current contribution by Schmidt et al.^12^ in this issue of ABC raises awareness and provides evidence to advocate assessing dynamic balance and mobility in old age patients with HFpEF. However, before this is implemented in clinical routine, their findings need to be strengthened in future studies.
